# High throughput screening aids clinical decision‐making in refractory acute myeloid leukaemia

**DOI:** 10.1002/cnr2.2061

**Published:** 2024-04-25

**Authors:** S. J. Jessop, N. Fuentos‐Bolanos, C. Mayoh, M. E. M. Dolman, G. Tax, M. Wong‐Erasmus, P. Ajuyah, V. Tyrell, G. M. Marshall, D. S. Ziegler, L. M. S. Lau

**Affiliations:** ^1^ Children's Cancer Institute Lowy Cancer Research Centre, UNSW Sydney Kensington New South Wales Australia; ^2^ Department for Haematology/Oncology Women's and Children's Hospital South Australia Australia; ^3^ Adelaide Medical School University of Adelaide South Australia Australia; ^4^ Kids Cancer Centre Sydney Children's Hospital New South Wales Australia; ^5^ School of Clinical Medicine UNSW Medicine & Health, UNSW Sydney Kensington New South Wales Australia

**Keywords:** acute myeloid leukaemia, case report, precision medicine, pre‐clinical, ruxolitinib

## Abstract

**Background:**

Despite advances in therapeutics for adverse‐risk acute myeloid leukaemia (AML), overall survival remains poor, especially in refractory disease. Comprehensive tumour profiling and pre‐clinical drug testing can identify effective personalised therapies.

**Case:**

We describe a case of ETV6‐MECOM fusion‐positive refractory AML, where molecular analysis and in vitro high throughput drug screening identified a tolerable, novel targeted therapy and provided rationale for avoiding what could have been a toxic treatment regimen. Ruxolitinib combined with hydroxyurea led to disease control and enhanced quality‐of‐life in a patient unsuitable for intensified chemotherapy or allogeneic stem cell transplantation.

**Conclusion:**

This case report demonstrates the feasibility and role of combination pre‐clinical high throughput screening to aid decision making in high‐risk leukaemia. It also demonstrates the role a JAK1/2 inhibitor can have in the palliative setting in select patients with AML.

## 1 INTRODUCTION

Despite advances in therapeutics for adverse‐risk acute myeloid leukaemia (AML), overall survival remains poor, especially in refractory disease. Comprehensive tumour profiling and pre‐clinical drug testing can identify effective personalised therapies. We describe the only case we are aware of where molecular analysis and in vitro high throughput drug screening (HTS) aided treatment decision making by suggesting resistance to conventional, yet toxic, treatment regimens and identified a tolerable, novel targeted therapy in a patient with ETV6‐MECOM fusion‐positive refractory AML. Ruxolitinib combined with hydroxyurea led to disease control and enhanced quality‐of‐life in a patient unsuitable for intensified chemotherapy or allogeneic stem cell transplantation (ASCT).

## 2 CASE

An 18‐year‐old girl was diagnosed with monosomy 7 AML with central nervous system involvement in March 2022 at Sydney Children's Hospital, New South Wales, Australia, after presenting with a haemoglobin of 68 g/L, platelet count of 67 × 10^9^/L and white cell count of 3.0 × 10^9^/L. The diagnosis was confirmed by bone marrow aspirate which showed a markedly hypercellular bone marrow with 57% myeloid blasts, and with flow cytometry confirmation of the myeloid lineage.

There was no relevant personal or family history, nor previous hospital presentations. Her disease was refractory to first‐line chemotherapy as per MyeChild^1^ protocol (Gemtuzumab 3 mg/m^2^ × 3 doses, cytarabine 100 mg/m^2^ × 20 doses, mitoxantrone 12 mg/m^2^ × 4 doses) and re‐induction FLAG‐Ida chemotherapy (fludarabine 30 mg/m^2^ × 5 doses, idarubicin 8 mg/m^2^ × 3 doses, cytarabine 2000 mg/m^2^ × 5 doses). Therapy was complicated by prolonged febrile neutropenia, refractory thrombocytopenia with recurrent hematemesis, *Candida glabrata* bacteraemia, *Klebsiella pneumoniae* sepsis, *Paenibacillus urinalis* central line‐associated bloodstream infection and pancreatitis, with a lipase that peaked at 500 U/L, along with deranged liver function tests.

To identify personalised therapeutic options, she was enrolled on the ZERO Childhood Cancer precision medicine study, which incorporates comprehensive tumour profiling (paired tumour‐germline whole‐genome sequencing and tumour whole‐transcriptomic sequencing)^2^ with preclinical drug testing.^3^ Molecular analysis identified *NRAS* G12S and *KRAS* G13D hotspot mutations and an ETV6‐MECOM fusion (Figure S1), not detected by diagnostic testing, and confirmed monosomy 7 (Figure S2). Whole‐transcriptomic sequencing detected high RNA expression of *MECOM* (consistent with ETV6‐MECOM fusion), *MCL1*, *JAK2*, *MAP2K1*, *PRKCD* and SRC family kinases (*FGR*, *HCK*, *LYN*) (Table S1).

**FIGURE 1:**
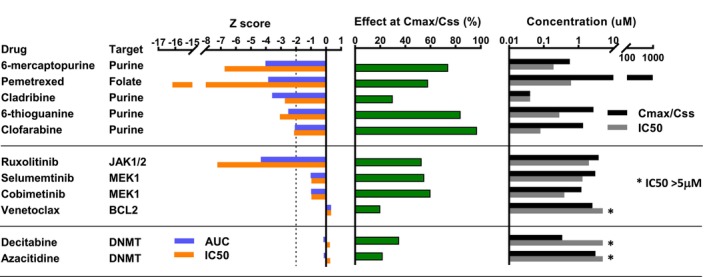
Line diagram demonstrating single‐agent high throughput screening results.

HTS was conducted in mononuclear cells (MNC) isolated from the patient bone marrow. Short tandem repeat profiling of MNC confirmed validity of cells and SNP microarray demonstrated 92% leukaemia cells. Cells were exposed to a library of 126 compounds, approved by the Food and Drug Administration (FDA) and/or Therapeutic Goods Administration (TGA) or in late pre‐clinical or experimental trial stages of development for childhood cancer.^4^ Drugs were tested in duplicate and added to achieve final concentrations of 0.5–5000 nM (10‐fold serial dilutions) for 72 h. A drug was considered a hit if the Z‐score of the AUC and IC50 was ≤ − 2. This represents high differential drug sensitivity compared to other samples in the drug database as AUC and IC50 are 2 standard deviations lower than the cohort mean.^3^ There were six drug hits: ruxolitinib (JAK1/2 inhibitor) and five anti‐metabolites (cladribine, clofarabine, 6‐thioguanine, 6‐mercaptopurine, pemetrexed) (Figure 1). Ruxolitinib was the only molecularly targeted drug hit in single‐agent HTS, consistent with high *JAK2* expression. The HTS showed in vitro resistance to venetoclax (BCL2 inhibitor), likely due to RAS mutations^5^, ^6^ and high *MCL1* expression.^7^, ^8^ Three in vitro drug combinations were tested based on molecular and HTS profiles and clinical agents commonly used in AML. Cells were treated for 72 h using a matrix design with five concentrations of each agent. Venetoclax was combined with ruxolitinib, trametinib or azacitidine, however no combination showed synergy (Table 1).

**TABLE 1:** In‐vitro drug testing with anti‐metabolites and targeted agents.

In vitro drug testing (single agent)							
Drug name	Drug mechanism	AUC *Z* score	Log2[IC50] *Z* score	IC50 (μM)	Cmax (μM)	Css (μM)	% Effect at MTD (%)
Cladribine	Purine analog	−3.59	−2.73	0.04	–	0.04	30
Clofarabine	Purine analog	−2.08	−2.13	0.08	1.36	–	97
6‐Thioguanine	Purine analog	−2.51	−3.07	0.28	0.52	2.7	84
6‐Mercaptopurine	Purine analog	−4.03	−6.75	0.19	0.56	–	74
Pemetrexed	Folate analog	−3.85	−16.17	0.61	985	–	58
Cobimetinib	MEK1/2 inhibitor	−0.99	−0.97	0.39	–	1.22	60
Selumetinib	MEK1/2 inhibitor	−1.02	−0.97	1.3	3.06	–	55
Venetoclax	BCL2 inhibitor	0.34	0.34	>5	–	2.51	20
Decitabine	DNMT inhibitor	−0.17	0.25	>5	0.34	–	35
Azacitidine	DNMT inhibitor	−0.15	0.28	>5	3.07	–	22
Ruxolitinib	JAK1/2 inhibitor	−4.34	−7.23	2.00	3.82	–	53

In vitro combination drug testing					
Tested combinations (DrugA + DrugB)	Drug targets (Drug A + DrugB)	Effect of DrugA at MTD (%)	Effect of DrugB at MTD (%)	Combined effect at the single agent MTD (%)	Observed drug interactions (Bliss score)
Venetoclax + ruxolitinib	BCL2 + JAK1/2	0	45	50	Synergy^a^
Venetoclax + trametinib	BCL2 + MEK1/2	0	25	30	Additive
Venetoclax + Azacytidine	BCL2 + DNMT	0	5	20	Additive

Abbreviations: %, percentage; AUC, area under the curve; Cmax, maximum concentration; Css, concentration at steady state; DNMT, DNA methyltransferase; IC50, half‐maximal inhibitory concentration; Log2, binary logarithm; MTD, maximum tolerated dose.

^a^ Synergy at higher dose levels. In the relevant range, additive drug–drug interaction was observed.

Due to her Eastern Cooperative Oncology Group (ECOG) performance score of 4, refractory disease and co‐morbidities, our patient was not a suitable candidate for further intensive salvage chemotherapy or ASCT. Based on the precision medicine results and use of ruxolitinib in post‐myeloproliferative neoplasm (MPN) AML, ruxolitinib (20 mg twice daily) was commenced with the intention to provide outpatient oral treatment, quality‐of‐life and slow disease progression. Hydroxyurea was prescribed at 2 g/day concurrently with the aim to reduce the white cell count (WCC) burden and limit complications of leucocytosis.

The patient's blast percentage and WCC remained elevated yet stable for two‐months (Figure S3). On commencing ruxolitinib, her packed red blood cell and platelet transfusion requirements decreased from an average of 7–2 and 21–10 units/month, respectively (Figure S3). Her quality‐of‐life, not formally assessed due to patient preference, and ECOG performance score of 2 significantly improved and she was discharged to outpatient services for the first time in 6 months. Unfortunately, she passed away from progressive disease 4 months later.

## 3 DISCUSSION

Patients with refractory adverse‐risk AML historically have poor response to conventional therapy, high morbidity and dismal long‐term outcomes.^9^ When ASCT is not suitable based on comorbidities, performance score, or patient preference, molecular sequencing and pre‐clinical drug testing may aid therapeutic decision‐making. This case used diagnostic features, comprehensive molecular profiling and single‐agent and combination in vitro HTS to aid a novel therapy decision in a patient with high‐risk AML, where severe toxicity limited conventional treatment options.

HTS has identified actionable targeted therapies in AML, which have impacted patient care, and along with molecular profiling, has aided the understanding of mechanisms of drug resistance.^10^, ^11^ An individualised systems medicine approach, using ex vivo drug sensitivity (DST) and resistance testing, has been developed to personalise AML therapy^10^ and led to clinical responses in three of eight patients.^10^ Another pilot study utilising an ex vivo platform in refractory AML observed a survival benefit when compared to patients treated as per physician recommendation (*n* = 12).^12^ All non‐DST‐guided patients had disease progression on therapy, where 75% displayed treatment responses when guided by DST results.^12^

The lack of in vitro sensitivity to venetoclax, somatic RAS mutations and high *MCL1* expression in our case strongly suggested clinical resistance to venetoclax. Without these findings, we may have considered therapy with venetoclax plus azacitidine and led to additional toxicity without clinical response.

ZERO demonstrated a rare ETV6‐MECOM fusion, more commonly found in therapy‐related AML^13^ and frequently co‐associated with monosomy 7.^14^, ^15^, ^16^ This fusion is associated with refractory disease to induction therapy^17^ and a dismal prognosis,^14^ supporting the high‐risk nature of our case. ETV6‐MECOM fusions are not directly druggable.

MAPK pathway activation was demonstrated by NRAS and KRAS hotspot mutations and high RNA expression of *MAP2K1*. Whilst potentially targetable with MEK inhibitors, monotherapy is ineffective in RAS‐mutant AML^18^ and this AML was resistant to trametinib in combination testing. There have been disappointing results when given in combination with venetoclax and azacitidine^19^ or cytarabine.^20^

JAK2 mutations are rare in de novo AML,^21^, ^22^ more commonly reported in post‐MPN AML.^23^ Whilst activation of JAK/STAT signalling in AML is thought to foster leukaemic proliferation,^24^ the frequency of high RNA expression of *JAK1*/*2* is unknown. One study reported high *JAK2* expression in M4/5 AML only.^25^

Ruxolitinib is an orally bioavailable JAK1/2 inhibitor, approved by the FDA for intermediate or high‐risk myelofibrosis and used in the setting of graft‐vs‐host disease and post‐MPN AML. High *JAK* RNA expression is not currently considered to be a biomarker of response to JAK1/2 inhibitors. JAK/STAT signalling inhibition using ruxolitinib has resulted in anti‐leukaemic activity in AML cell lines in vitro, yet wasn't replicated in patient derived xenograft models.^24^ In JAK‐mutated post‐MPN AML, ruxolitinib demonstrated anti‐leukaemic activity,^26^ and in the palliative setting, led to spleen size reduction, improvement in constitutional symptoms and quality‐of‐life.^27^ Whilst tolerable, there have been no sustained objective responses seen in the curative or palliative setting in adult or paediatric AML.^28^, ^29^, ^30^

Whilst improved overall survival has been demonstrated with ASCT as compared to salvage chemotherapy in refractory AML, our patient exhibited all five adverse pre‐transplantation variables as established by Duval et al, including a lack of complete remission, circulating blasts, lack of availability of a non‐human leukocyte antigen‐identical sibling donor, poor performance score and poor‐risk cytogenetics.^31^ Ruxolitinib in our patient was tolerable, improved quality‐of‐life and reduced blood transfusion requirements substantially, leading to time out of hospital. Whilst not curative, it prolonged life compared to that expected with supportive care alone.^32^ This cannot be explained by the concurrent use of hydroxyurea, with cytoreduction shown not to lead to differences in short or long‐term outcomes in newly diagnosed AML.^33^ The molecular and HTS results directed this novel treatment decision and avoided what may have been a toxic and unsuccessful therapy regimen.

Our case describes a particularly biologically and cytogenetically high‐risk patient, where toxicity limited therapeutic options, and demonstrates how molecular analysis and pre‐clinical testing can aid tailored, clinically implementable treatment choices.

## AUTHOR CONTRIBUTIONS

**S. J. Jessop:** Conceptualization (lead); formal analysis (lead); writing – original draft (lead); writing – review and editing (lead). **N. Fuentos‐Bolanos:** Writing – original draft (supporting); writing – review and editing (supporting). **C. Mayoh:** Data curation (supporting); writing – review and editing (supporting). **M. E. M. Dolman:** Data curation (supporting); writing – review and editing (supporting). **G. Tax:** Data curation (supporting); writing – review and editing (supporting). **M. Wong‐Erasmus:** Data curation (supporting). **P. Ajuyah:** Data curation (supporting). **V. Tyrell:** Data curation (supporting). **G. M. Marshall:** Writing – review and editing (supporting). **D. S. Ziegler:** Writing – review and editing (supporting). **L. M. S. Lau:** Conceptualization (equal); data curation (equal); formal analysis (equal); supervision (supporting); writing – original draft (supporting); writing – review and editing (supporting).

## CONFLICT OF INTEREST STATEMENT

All authors of this publication are employed by the Children's Cancer Institute at UNSW Sydney and proudly work for the ZERO Childhood Cancer precision medicine study.

## ETHICS STATEMENT

This manuscript has been prepared in accordance to the Committee on Publication Ethics (COPE) guidelines. It has been performed in an ethical and responsible way, with no research misconduct, plagiarism or unethical research.

## Supporting information


**Figure S1.** Arriba plot demonstrating ETV6‐MECOM fusion.

**Figure S2.** Circos plot confirming presence of monosomy 7.

**Figure S3.** Demonstration of patient blood levels and product requirements at commencement of MyeChild chemotherapy, FLAG‐Ida re‐induction and precision medicine guided ruxolitinib and hydroxyurea.

**Table S1.** Patient molecular results generated from whole genome sequencing and tumour whole transcriptomic sequencing.

## Data Availability

Data are available on request to the corresponding author.
