# Supplementary material for: High throughput screening aids clinical decision‐making in refractory acute myeloid leukaemia
Source: Cancer Rep (Hoboken). 2024 Apr 25;7(4):e2061. doi: 10.1002/cnr2.2061 (PMC11044912; doi:10.1002/cnr2.2061)
Supplement: Supplementary file 1 — Figure S1. Arriba plot demonstrating ETV6‐MECOM fusion. [file CNR2-7-e2061-s002.pdf]

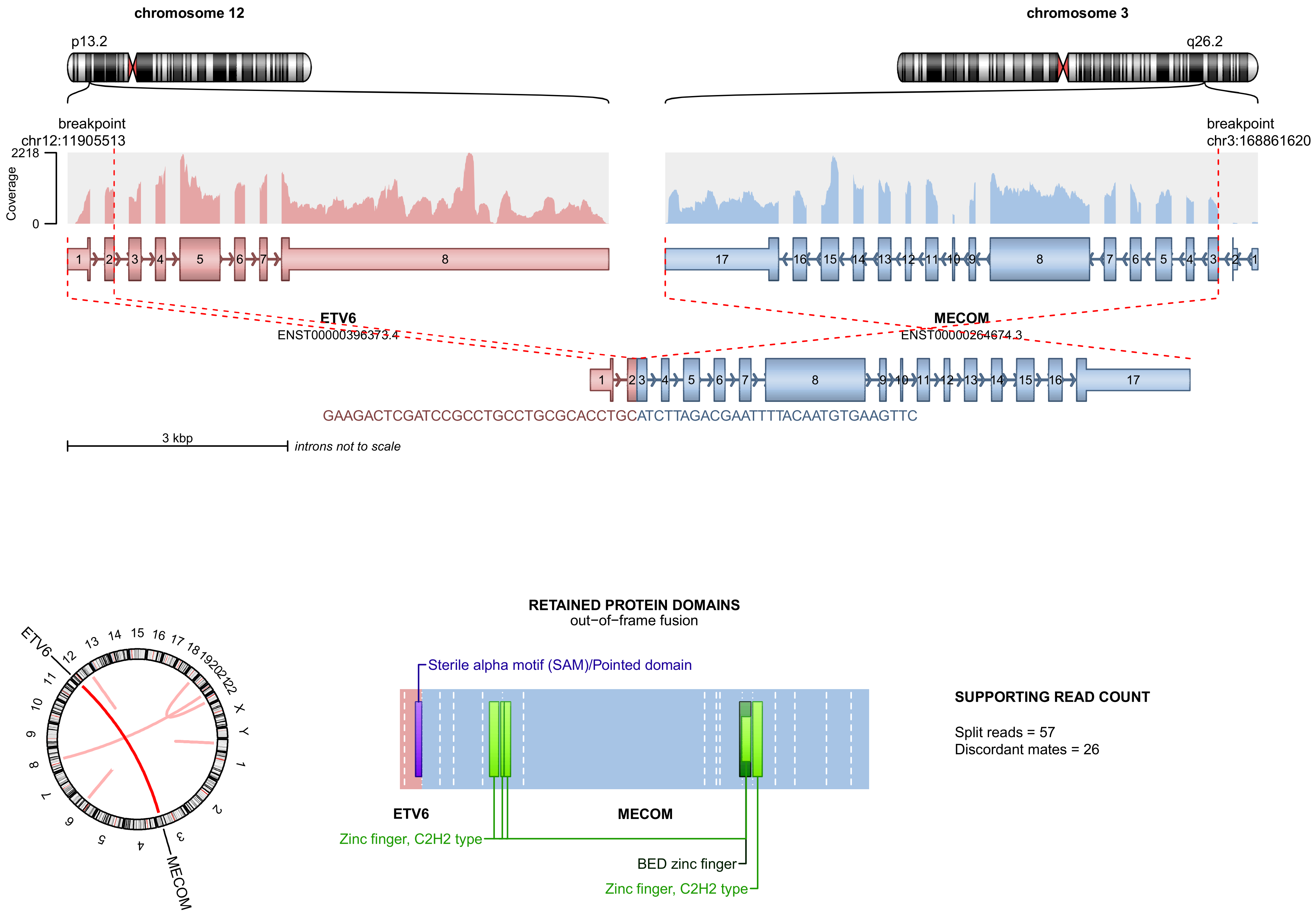

chromosome 12
chromosome 3
p13.2
q26.2
breakpoint
chr12:11905513
breakpoint
chr3:168861620
Coverage
2218
0
ETV6
ENST00000396373.4
MECOM
ENST00000264674.3
GAAGACTCGATCCGCCTGCCTGCGCACCTGCATCTTAGACGAATTTTACAATGTGAAGTTC
3 kbp
introns not to scale
RETAINED PROTEIN DOMAINS
out-of-frame fusion
Sterile alpha motif (SAM)/Pointed domain
ETV6
MECOM
Zinc finger, C2H2 type
BED zinc finger
Zinc finger, C2H2 type
SUPPORTING READ COUNT
Split reads = 57
Discordant mates = 26
