# Supplementary figures and images for: High throughput screening aids clinical decision‐making in refractory acute myeloid leukaemia
Source: Cancer Rep (Hoboken). 2024 Apr 25;7(4):e2061. doi: 10.1002/cnr2.2061 (PMC11044912; doi:10.1002/cnr2.2061)

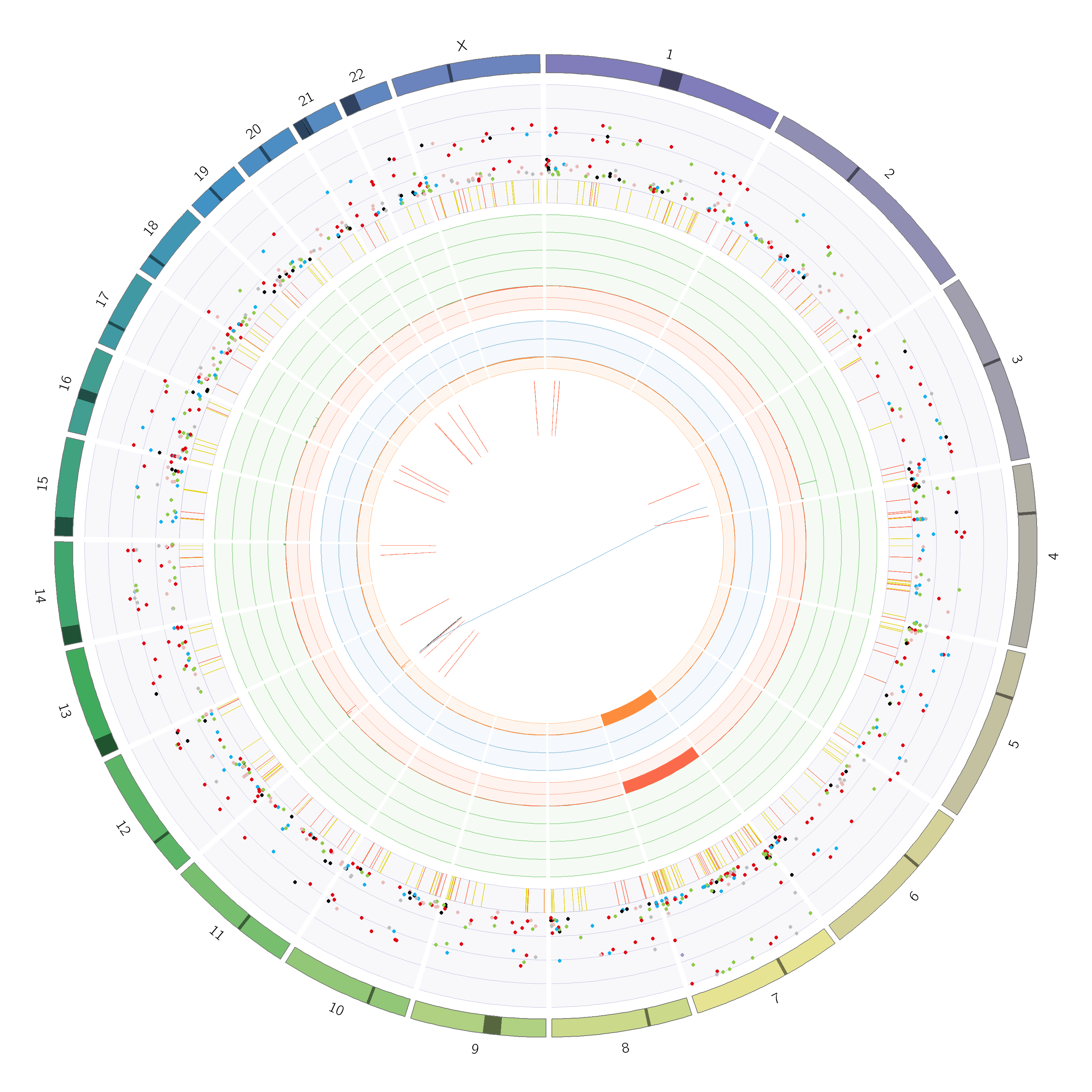

Supplement: Supplementary file 2 — Figure S2. Circos plot confirming presence of monosomy 7. [file CNR2-7-e2061-s004.png]

## Slide 1
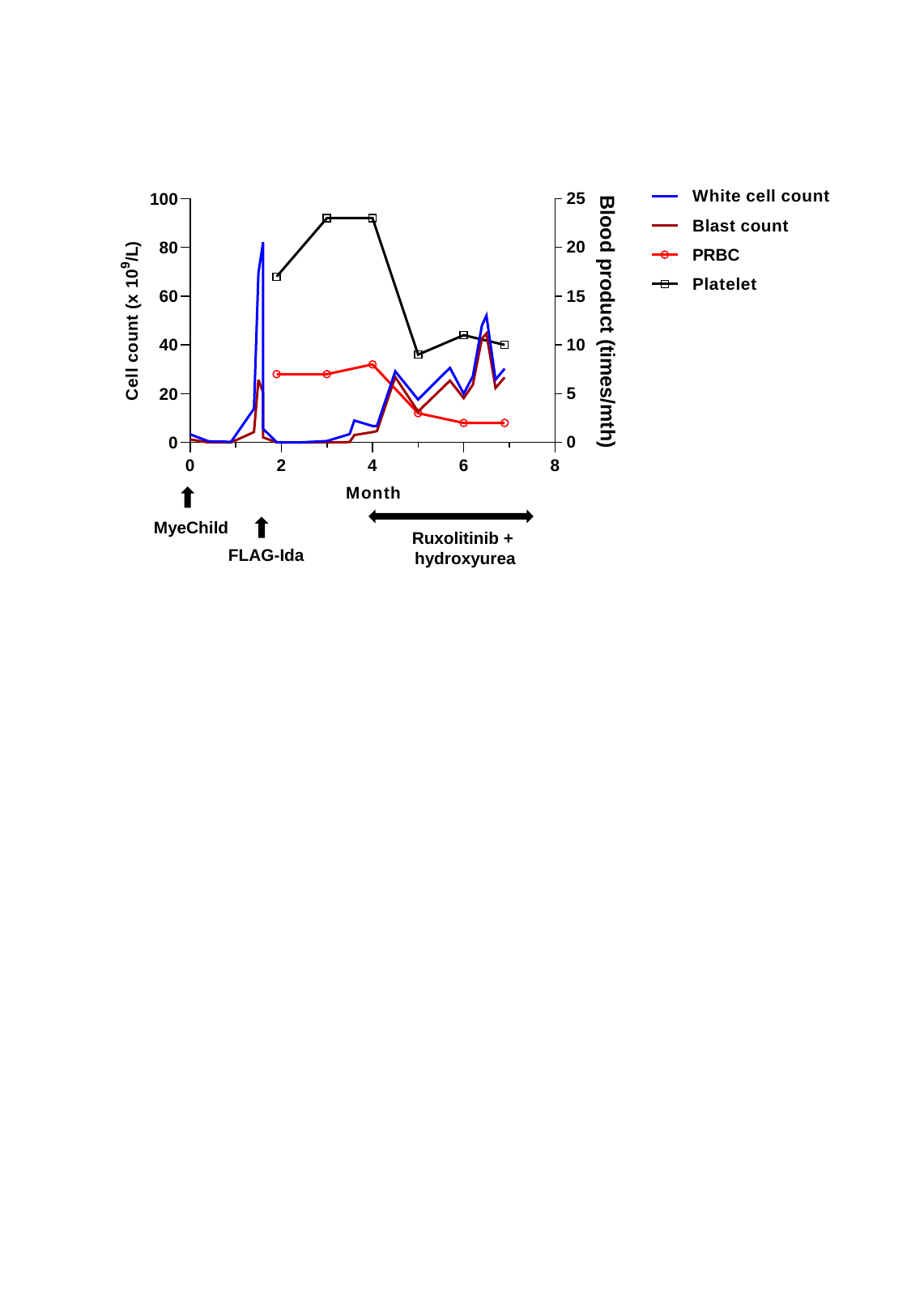

MyeChild
Ruxolitinib +
hydroxyurea
FLAG-Ida

Supplement: Supplementary file 3 — Figure S3. Demonstration of patient blood levels and product requirements at commencement of MyeChild chemotherapy, FLAG‐Ida re‐induction and precision medicine guided ruxolitinib and hydroxyurea. [file CNR2-7-e2061-s003.pptx]
