# Supplementary material for: High throughput screening aids clinical decision‐making in refractory acute myeloid leukaemia
Source: Cancer Rep (Hoboken). 2024 Apr 25;7(4):e2061. doi: 10.1002/cnr2.2061 (PMC11044912; doi:10.1002/cnr2.2061)
Supplement: Supplementary file 4 — Table S1. Patient molecular results generated from whole genome sequencing and tumour whole transcriptomic sequencing. [file CNR2-7-e2061-s001.docx]

| **Pathway** | **Gene** | **Aberration** | **FC** | **Z-score** |
| --- | --- | --- | --- | --- |
| TGF-B signalling | MECOM | ETV6-MECOM fusion | - | - |
|  |  | High RNA expression | 5.46 | 1.09 |
| MAPK | NRAS | G12S het mutation | - | - |
|  | KRAS | G13D het mutation | - | - |
|  | MEK2 | High RNA expression | 3.87 | 2.01 |
| DNA repair | MCL1 | High RNA expression | 6.01 | 2.42 |
| JAK/STAT | JAK2 | High RNA expression | 8.86 | 3.20 |
| SRC family kinases | FGR | High RNA expression | 19.52 | 2.66 |
|  | HCK | High RNA expression | 20.40 | 2.69 |
|  | LYN | High RNA expression | 10.46 | 2.34 |

FC: fold copy change; het: heterozygous

Table 1. Patient molecular results generated from whole genome sequencing and tumour whole transcriptomic sequencing
